# Screen Time and Associated Factors in Early Adolescent Age Group Experience From a North Indian Center

**DOI:** 10.7759/cureus.85878

**Published:** 2025-06-12

**Authors:** Sanghamitra Ray, Euden Bhutia, Rajesh Kumar Meena

**Affiliations:** 1 Pediatrics, Vardhman Mahavir Medical College and Safdarjung Hospital, Delhi, IND; 2 Pediatrics, Dr. Baba Saheb Ambedkar Medical College and Hospital, Delhi, IND; 3 Pediatrics, University College of Medical Sciences, Delhi, IND

**Keywords:** academic performance, adolescent, media exposure, screen time, sleep duration

## Abstract

Background

In recent years, the rise of digital technology has led to a significant increase in screen time among children, raising concerns about the potential effects of excessive screen exposure on children's development. The objectives were to estimate screen exposure and media use in the early adolescent age group and to correlate the exposure with demographic characteristics and its effect, if any, on school performance and sleep duration.

Material and methods

Children aged between 10 and 14 years attending the Pediatric Outpatient Department were enrolled. Data collection was done via interview based on a structured, pre-tested questionnaire. Screen time and media exposure of the adolescent and parents were assessed, and their effect on sleep duration, academic performance, and outdoor activity was noted.

Results

Girls had significantly less screen time than boys. Socio-economic status and literacy level of parents failed to show a significant association. The screen time of parents had a significant impact on the duration of screen time in adolescents. Screen time failed to show any effect on sleep duration and school performance. Playtime was significantly less in the group with higher screen time. We could not find any effect of screen time on academic performance or sleep duration in this study.

## Introduction

Nowadays, our society is undergoing a major change, as with each passing day, our dependency on electronic devices and media is increasing exponentially, and this has greatly improved our access to the newer information and ideas. As we are becoming more and more dependent on electronic media, it is probably the children and adolescents who are bearing the brunt of the situation most. Excessive exposure to multimedia from very early in life is indeed becoming a new health hazard. Unsupervised exposure to age-inappropriate media content is a matter of great concern as it affects the growing minds negatively [1,2].

“Screen time” refers to the time spent by an individual on electronic devices and media for activities such as online studies, watching television, entertainment purposes, etc. Studies have documented the negative impact of excessive media exposure and screen time in the form of decreased outdoor physical activity, reduced sleep duration, sleep disorders, behavioral abnormalities, poor scholastic performance, and delinquent behavior in different age groups [1,2]. In 2016, American Academy of Pediatrics (AAP) released guidelines regarding screen time exposure in children, and also emphasized the importance of parent-child shared media use such as watching educational programs or playing video games together, can strengthen parent-child bonds and restricts screen timing ensuring that the child takes part in other developmentally healthy activities [3]. Adolescents are more likely to see adult-directed media content, and if allowed unsupervised media time, can lead to exposure to violent, obscene content, affecting their mental development and behavior. Sleep health of adolescents has been adversely affected, and both the delayed sleep onset and reduced sleep duration have been documented because of excessive media use [4,5]. Limited literature is available from low- and middle-income countries (LMIC), including India, and most of these studies focused on children below five years of age. A previous study documented a high screen time among Indian adolescents [6]. In this study, we aimed to estimate the average screen time, delineate the associated demographic factors, and evaluate the impact of screen time on the sleep pattern and academic performance of the early adolescent age group.

## Materials and methods

This was a cross-sectional study conducted in the Department of Pediatrics at a tertiary care teaching hospital in North Delhi, primarily catering to the children and adolescents belonging to urban low- and middle-income families. Approval from the institutional ethics committee was obtained before the commencement of the study. Written informed consent was taken from the accompanying parent(s)/guardian of children, and verbal assent was also taken from participants above seven years of age. All children aged between 10 and 14 years attending the Pediatric Outpatient Department of the hospital between November 2019 and April 2020 were approached and enrolled. All enrolled children were interviewed based on a structured, pre-tested questionnaire. After taking informed consent, the patient’s self-reported questionnaire was filled out by one of the investigators. A face-to-face interview was conducted in a separate room with confidentiality maintained. To avoid inter-observer variation, all participants were interviewed by the same investigator. Critically sick children, children with developmental delay, behavioral disorders like autistic spectrum disorders, attention deficit hyperactivity disorder, and those with hearing or language deficit were excluded from the study. 

Relevant demographic details, including the number of siblings, education of parents, number of rooms at the house, etc., were documented in a pre-designed proforma. Socio-economic status was determined using the modified Kuppuswamy scale [7]. Screen time and media exposure were assessed by documenting the use of personal mobile phone, personal computer, television, tablets, and other gadgets. The total number of smartphones at home, use of media devices at meal time, during homework, in the washroom, and before sleeping were taken into account while calculating the total screen time. The screen time of parents at home was also noted. Outdoor physical activity of the adolescent, along with academic performance at school, was documented. Use of social media with or without parents’ knowledge was recorded in each case. 

Statistical analysis

Data were entered in a Microsoft Excel (Microsoft® Corp., Redmond, WA) sheet and were analyzed using IBM Statistical Package for Social Sciences (SPSS) for Windows (IBM Corp., Version 23, Armonk, NY). Continuous data were summarized as mean ± SD, while categorical data were presented in numbers and percentages. For determining the factors associated with increased screen time, proportions and means (SD) or medians (IQR) were compared between children having screen time <2 hours/day and >2 hours/day, by univariate analysis using the chi-square test and odds ratios. Multiple logistic regression was performed using screen time as a dependent variable and factors with P < 0.30 on univariate analysis and factors which has direct plausibility as independent variables. P-value < 0.05 was considered statistically significant.

## Results

**Figure 1:**
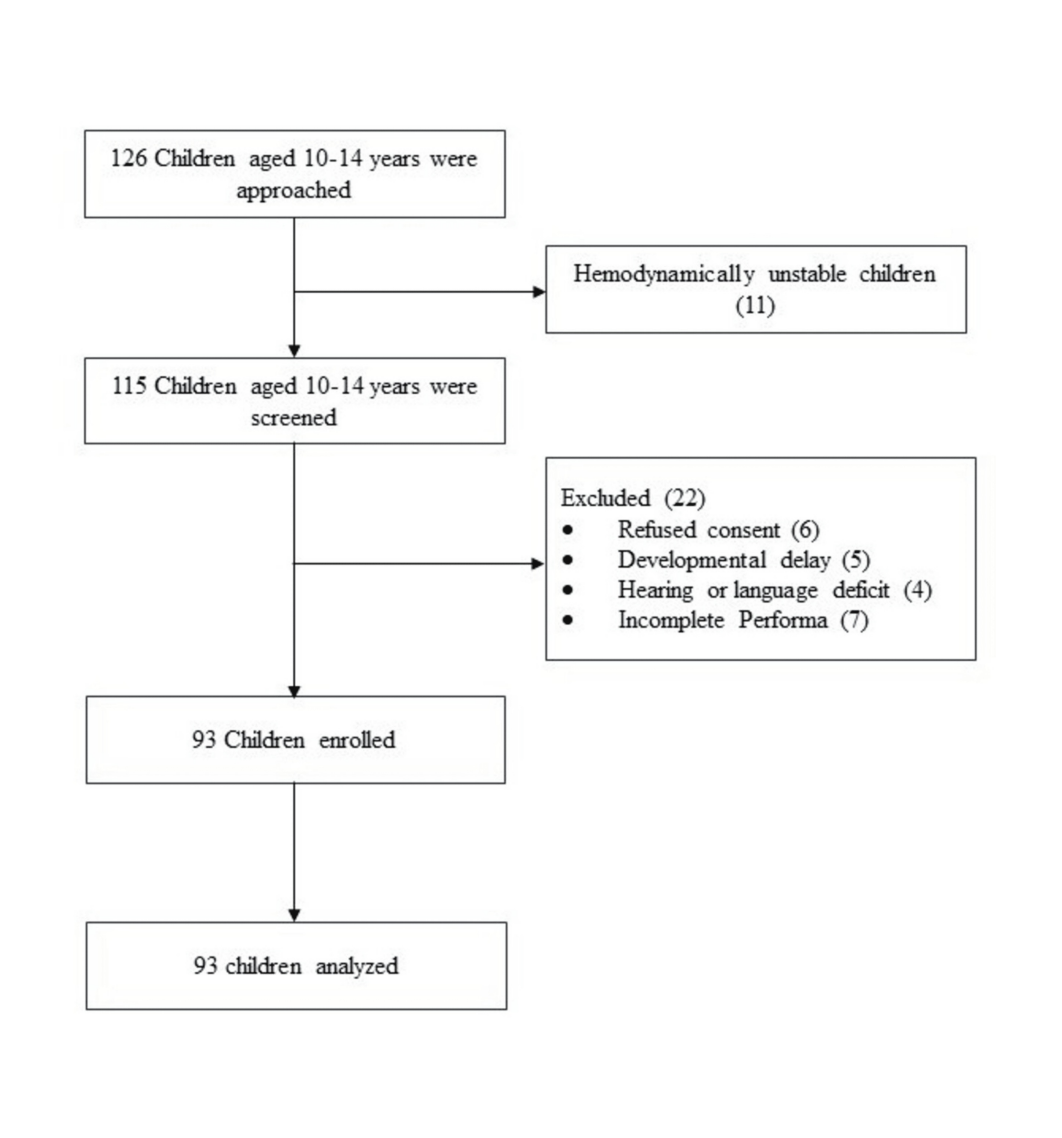
Inclusion flowchart for cases in the study

In total, 115 children in the 10-14 year age group from the Pediatric Outpatient Department were approached and screened for enrolment. Finally, 93 children of the early adolescent age group were enrolled in the study after excluding 23 children (Figure 1).

The mean (SD) age of the participants was 11.4 (0.995) years; 67% of them were between 10 and 12 years. One-third of the participants were studying in the sixth standard, and another 26% were in the fifth standard. The majority of the participants have one or more siblings, while 3.2% were single children. The majority (76%) of the children were from lower and upper-lower socio-economic backgrounds as per the modified Kuppuswamy classification, and 59% had two or more rooms in their house. One-fifth of the fathers and 35.5% of mothers were illiterate. The baseline characteristics of the participants are shown in Table 1.

**Table 1:** Demographic variables and baseline characteristics of the study cohort (n = 93) Values expressed as number (percentage %). *Mean ± SD

Parameter		n (%)
School performance in last examination	>50% marks	60 (64.5%)
	<50 % marks	33 (35.5%)
Education of the father	Illiterate	17 (18%)
	Primary	23 (25%)
	Secondary	34 (37%)
	Higher secondary and above	13 (14%)
	Graduate and above	6 (6.5%)
Education of the mother	Illiterate	33 (35.5%)
	Primary	23 (25%)
	Secondary	24 (26%)
	Higher secondary and above	7 (7.5%)
	Graduate and above	6 (6.5%)
Socio-economic status	Lower	35 (37.6%)
	Upper lower	36 (38.7%)
	Lower middle	17 (18.3%)
	Upper middle	5 (5.4%)
	Upper	Nil
Outdoor activity (days/week)	No outdoor activity	17 (18.2%)
	<3/week	11 (11.8%)
	3-6 days/week	31 (33.3%)
	Every day	34 (36.5%)
Number of smartphones at home	Nil	8 (8.5%)
	1	47 (50.5%)
	≥2	38 (41%)
Screen time (hours/day)	≤​​​​​​​2	59 (63%)
	>2	34 (37%)
Playtime/day (hours)*	1.16 ± 0.97	-
Screen time/day*	2.06 ± 1.34	-
Sleep duration (hours)*	8.5 ± 0.95	-
Screen time of mother at home (hours/day)*	0.77 ± 0.98	-
Screen time of father at home (hours/day)*	0.83 ± 0.96	-

The average screen time of the cohort was 2.06 (1.34) hours per day, and 37% had daily screen exposure of more than two hours. Screen time of six hours/day was documented in one case, while 10% of children reported that they did not watch the screen at all. Screen time of females was significantly lower than that of male children (P < 0.009). Almost 67% of children used to watch the screen while taking meals. Only 14% used screens or media for doing their daily homework. Very few of them (2.2%) had a group chat for daily homework; 64.5% of adolescents achieved more than 50% marks in the last annual examination at school. Approximately two-thirds of the children had a television installed (57%) in their bedroom, and a similar number had exposure (59%) to any type of electronic media before sleep. No child used the media while using the washroom. One-third of children had television continuously running in the background while doing usual household work. Only one child had a personal gadget (mobile phone), while social media was used individually by two children. Age and class of study were not found to be associated with variation in screen time. One-fifth of the participants did not have any outdoor activity, while another 23% and 37% reported outdoor activity on three days and seven days/week, respectively.

On comparing the children with ≤2 hours daily screen time with those having >2 hours of daily screen time no significant difference was found in terms of number of smart phones at home, number of siblings of the child, number of rooms in the house, parental education status, and socio-economic status of the family (Table 2).

**Table 2:** Comparison of children with screen time less than two hours per day with those having more than two hours per day Values expressed as number (percentage %). ^a^Mean (SD)

Parameters	Children with screen time ≤2 hours/day (n = 59)	Children with screen time >2 hours/day (n = 34)	p-value
Age (years)^a^	11.55 (1.041)	11.065 (0.8341)	0.21
Female	31	8	0.009
School performance in the last examination (>50% marks )	34	26	0.076
Number of siblings (≥2)	35	16	0.285
Number of rooms (≥​​​​​​​2)	33	22	0.512
Education of father (illiterate or primary)	25	15	1.000
Education of mother (illiterate or primary)	38	18	0.379
Socio-economic status (lower)	46	25	0.623
Outdoor activity (<4 days/week)	29	19	0.667
Number of smartphones at home (≥​​​​​​​2)	24	14	1.00
Screen watching during mealtime	30	33	0.001
Screen exposure before sleep	31	24	0.125
Presence of screen in bedroom	30	23	0.133
Screen time (hours/day)^a^	1.22 (0.733)	3.515 (0.7928)	<0.001
Playtime (hours/day)^a^	1.364 (1.0781)	0.809 (0.6156)	0.007
Sleep duration (hours/day)^a^	8.432 (0.9119)	8.662 (1.0054)	0.243
Screen time mother (hours/day)^a^	0.585 (0.9521)	1.103 (0.9675)	0.014
Screen time father (hours/day)^a^	0.661 (0.7793)	1.118 (1.1746)	0.027

Though screen exposure before sleep was high in both groups (59% in the whole study population), the difference among groups was not significant. However, children with increased screen time had screen exposure even during mealtime, which was statistically significant (P < 0.001). The playtime of children with higher screen time was significantly less compared to the other group (P < 0.007). Television running in the background while doing other work also did not contribute to increased screen time. One of the significant findings of this study was that the screen time of mothers and fathers was significantly higher in the group where children had higher screen time (P < 0.014 and P < 0.027, respectively). We could not find the effect of screen time on school performance and sleep duration.

In univariate analysis, among the risk factors evaluated, parental screen time had a significant association with prolonged screen time. On multivariable logistic regression, the adjusted OR for prolonged screentime was higher (>1) for the number of rooms, low education status of the mother, and screen time of father and mother, but these were not statistically significant (Table 3).

**Table 3:** Association of risk factors and screen time >2 hours

Variables	OR	95% CI	Adjusted OR	95% CI
Sex	0.278	0.108, 0.714	0.361	0.132, 0.985
Siblings	0.610	0.260, 1.427	0.583	0.183, 1.859
Number of rooms	0.931	0.398, 2.182	1.439	0.420, 4.939
Education of the father	0.931	0.398, 2.182	0.534	0.150, 1.902
Education of the mother	1.608	0.681, 3.796	1.314	0.352, 4.899
Socio-economic status	1.274	0.478, 3.393	1.080	0.274, 4.263
Screen in the bedroom	0.495	0.205, 1.194	0.553	0.195, 1.570
Screentime of the mother	3.800	0.658, 21.955	1.899	0.268, 13.445
Screentime of the father	10.000	1.116, 89.604	8.026	0.794, 81.177

## Discussion

AAP recommends that children below two years should not be exposed to screens at all, while for those more than two years, screen time should be restricted to less than two hours per day [8]. In 2021, the Indian Academy of Pediatrics published a Guideline on Screen Time and Digital Wellness in Infants, Children, and Adolescents, where the group recommended that screen time must not replace other activities such as outdoor physical activities, sleep, family and peer interaction, and studies [9]. Families should ensure a nurturing, supportive, and secure environment at home, and parents/caregivers should ensure that the content being watched is educational, age-appropriate, and non-violent. In the IAP guideline, no clear cut-off for screen time was mentioned for adolescents of 10-18 years of age, but it was emphasized that developmental activities should not be hampered. These activities include at least one hour of outdoor physical activity, eight to nine hours of night-time sleep, and time for schoolwork, meals, hobbies, peer interaction, and family time. In case of compromise on these activities due to screen time, then screen time needs to be appropriately reduced, and parental supervision was also highlighted [9].

In the current study involving 93 children of the early adolescent age group (10-14 years), the average screen time duration is more than that recommended by the guidelines. The girls had significantly less screen time compared to their male counterparts. Among the participants, a significant proportion of children have screen exposure while having their meals and before sleep, and do not have any outdoor physical activity. The average screen time of parents of children with higher screen time is also high. The lesser screen time of girls can be attributed to the fact that boys are given more privileges than girls, and girls are often given additional responsibilities of household work, which may be the cause of their reduced screen time. The screen time of parents significantly affected the screen time of the children. Among adolescents, higher duration of screen time has been associated with reduced physical activity, abnormal behavioral conduct, and poorer mental health status. Among adolescents, TV viewing is the most studied sedentary behavior [10,11].

Self-reported screen time is consistently higher in boys than girls, as in our study [12,13]. Reported daily screen time appears to increase with age [12], though we could not find such an association. A recent systematic review with meta-analysis by Schaan et al. [14] showed a high prevalence of excessive screen time and TV viewing among Brazilian adolescents and also reinforced the need to monitor and identify the high-risk adolescent groups. Excessive screen time is also considered one of the predisposing factors in causing non-communicable diseases [15] and increasing morbidity in adult life [16].

As most of the children are from poorer section of the society and studied in government schools, very few used mobiles or other gadgets for homework purpose or had school group chat, for the same studies have shown that with increasing age of the child there is sharp increase in screen time and media exposure though our study could not find such association [17,18]. Adolescence is a critical period of development during which important aspects of health and well-being are easily affected by external influences. As electronic media use among adolescents is increasing significantly, the potential relationship between screen time and adolescent well-being is of utmost importance. The most important markers of adolescent well-being that are affected are mainly the academic performance [19], sleep quality and duration, and peer interactions [20]. In our study, we did not evaluate peer interaction, but academic performance and sleep duration were not found to be related to screen time. The negative association between screen time and academic performance has been found in studies [21]. We assume that more time dedicated to media use restricts the usual allotted time spent on studying. Our research also showed a significant reduction in playtime in those with higher screen exposure. While some research has found associations between excessive screen time and lower academic achievement, other studies have failed to establish a clear link, as in our study. A recently published literature review analyzing the association between youth screen media use and sleep found an association between screen media use and delayed bedtime and/or decreased total sleep time in 90% of the included studies. Psychological stimulation, light exposure, reduced secretion of melatonin, and increased physiological alertness have been implicated as causative factors [22].

Three-fourths of adolescents in the USA report the availability of at least one screen-media device in their bedroom, with roughly 60% reporting regular use of these devices during the hour before bedtime [23,24].

Media devices in the bedroom were more common in lower-income youth (68% versus 37%). The authors speculated that the higher rate of electronic gazettes in the bedrooms of adolescents from lower-income families may be the result of more frequent room sharing, sleeping in a multi-purpose room, or differing family preferences [25].

Most of the recent studies analyzed total screen time per day as a predictor, but many more effects on sleep have been documented with media use in the bedroom around bedtime [26,27].

Children having parents with a lower level of education had more screen time at home compared with children whose parents were more educated [28]. As per a systematic review nine out of 10 studies reported a positive association between parents’ TV watching and children’s screen time and parents’ own sedentary lifestyle was also linked to children’s screen time as we could find in our study which is directly attributed to the parental influence on the behavior of an adolescent [29].

Strength of the study

The sample size is good, and parental screen time has been taken into consideration. Most of the Indian studies focused on the under-five age group. Very few studies have looked into the screen time of children from lower strata.

Limitations of the study

Being a hospital-based study, the results cannot be generalized. Some of the variables, such as adolescent behavioral anomalies, psycho-somatic disorders, and peer interactions, were not taken into account.

## Conclusions

The relationship between screen time and children's well-being is complex and multifaceted in nature. Excessive screen time has been associated with male sex, increased parental screen time, and significantly reduced playtime in the studied early adolescent age group. Further research is needed to better understand the mechanisms underlying the impact of screen time on children and to develop evidence-based guidelines for promoting healthy screen use habits in childhood and adolescence.
